# Nuclear Cogeneration of Methanol and Acetaldehyde from Ethylene Glycol Using Ionizing Radiation

**DOI:** 10.1021/acs.iecr.3c03317

**Published:** 2023-12-04

**Authors:** Arran George Plant, Bor Kos, Anže Jazbec, Luka Snoj, Malcolm John Joyce, Vesna Najdanovic-Visak

**Affiliations:** † School of Engineering, Lancaster University, Lancaster LA1 4YW, U.K.; ‡ Jožef Stefan Institute, Ljubljana 1000, Slovenia; § Chemical Engineering and Applied Chemistry (CEAC), Energy & Bioproducts Research Institute (EBRI), Aston University, Birmingham B4 7ET, U.K.

## Abstract

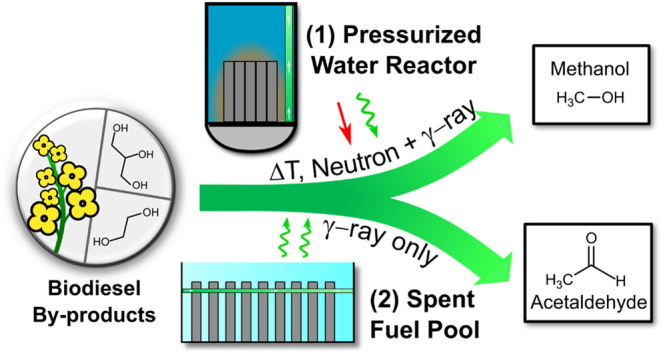

Despite offering low-carbon and reliable energy, the utilization of nuclear energy is declining globally due to high upfront capital costs and longer returns on investments. Nuclear cogeneration of valuable chemicals from waste biomass-derived feedstocks could have beneficial impacts while harnessing the underutilized resource of ionizing energy. Here, we demonstrate selective methanol or acetaldehyde production from ethylene glycol, a feedstock derived from glycerol, a byproduct of biodiesel, using irradiations from a nuclear fission reactor. The influence of radiation quality, dose rate, and the absorbed dose of irradiations on radiochemical yields (*G*-value) has been studied. Under low-dose-rate, γ-only radiolysis during reactor shutdown rate (<0.018 kGy min^–1^), acetaldehyde is produced at a maximum *G*-value of 8.28 ± 1.05 μmol J^–1^ and a mass productivity of 0.73 ± 0.06% from the 20 kGy irradiation of neat ethylene glycol. When exposed to a high-dose-rate (6.5 kGy min^–1^), 100 kGy mixed-field of neutron + γ-ray radiations, the radiolytic selectivity is adjusted from acetaldehyde to generate methanol at a *G*-value of 2.91 ± 0.78 μmol J^–1^ and a mass productivity of 0.93 ± 0.23%. Notably, utilizing 422 theoretical systems could contribute to 4.96% of worldwide acetaldehyde production using a spent fuel pool γ-ray scheme. This research reports *G*-values and production capacities for acetaldehyde for high-dose scenarios and shows the potential selectivity of a nuclear cogeneration process to synthesize chemicals based on their irradiation conditions from the same reagent.

## Introduction

Nuclear power is a low-carbon source of electricity with a carbon output of 12 gCO_2_-eq kWh^–1^, which is only surpassed by intermittent, volatile wind at 11 gCO_2_-eq kWh^–1^.^1^ Despite this, the high capital costs and slower return on investment associated with nuclear power plants have led to a relative decline in the global share output of nuclear electricity by source, from 17% in 2000 to 10% in 2021.^2^ Techno-economic assessments have shown that nuclear cogeneration of higher-valued chemicals can increase the economic prospects of large nuclear power plants without negatively affecting electricity output.^3,4^ While planned future cogeneration Gen-IV systems aim to incorporate hydrogen gas cogeneration alongside electricity,^5,6^ it has been shown that the low value of hydrogen gas provides negligible financial benefits.^3,7−10^ Chemical coproducts such as propylene from propane have been proposed to improve the internal rate of return (IRR) of investment to approximately 8%.^3^ However, harnessing the underutilized energy available in the ionizing radiation yield from nuclear processes to initiate unique radiation-directed chemical reactions could yield more profitable and useful applications in chemical synthesis without the requirement for energy-intensive processes and conventional catalysts.^11^ Additionally, a greater focus on bioderived feedstocks to generate value-added chemicals could alleviate the reliance on what can be limited petrochemical feedstocks.

One such bioderived chemical feedstock, refined glycerol, has a notable sustainability issue due to global production excesses (∼500,000 kt yr^–1^) that are currently directed toward low-value applications such as incineration and animal feed.^12^ Glycerol has the potential as a bioderived platform chemical for the synthesis of valuable chemicals, such as glycerol carbonates, epichlorohydrin, and solketal.^13^ Additionally, glycerol can be converted to ethylene glycol through high-throughput hydrogenolysis processes which expands the scope for radiolytically synthesized products derived from renewable glycerol.^14,15^ Two valuable products that can be derived from glycerol are acetaldehyde and methanol. Acetaldehyde is generated industrially from petroleum-derived ethylene via the Wacker process using expensive palladium catalysts at a worldwide production capacity of ∼1.3 × 10^6^ tonnes per year as of 2021.^16,17^ Acetaldehyde is an important platform chemical for producing peracetic acid, pyridine bases, and pentaerythritol.^18^ Methanol is currently synthesized from natural gas via steam reforming processes, contributing to a large worldwide production capacity of ∼1.1 × 10^8^ tonnes per year.^19^ Although the catalyst-free radiolytic production of these compounds from ethylene glycol has been reported,^20,21^ little consideration has been given to industrial implementation optimization of the reaction parameters for radiolytic synthesis.

Radiation chemical yields or *G*-values have been reported widely in the radiolysis literature to assess the effectiveness of a radiolytic transformation to either a reactive transient species (lower-case *g*-value) or molecular products (upper-case *G*-value). Historically, *G*-values were expressed in the units of 100 molecules per eV (100 eV^–1^), but modern SI unit convention adopts micromoles per joule (μmol J^–1^). The conversion factor between these units is 1 molecule per 100 eV to 0.1036 μmol J^–1^. Importantly, many reports quote radiolytic product *G*-values from organics in heavily diluted aqueous samples irradiated with small or near-zero absorbed doses (typically <1 kGy), consequently generating small product concentrations proportional to the entire irradiated sample. If such conditions were industrially considered, huge volumes of reaction media would need to be recycled and processed which would be wasteful and costly. Using larger doses and higher concentrations presents a more realistic case for higher yields and resource utilization. Since product *G*-values decrease with increased absorbed doses for most organic systems and specifically for ethylene glycol radiolysis,^20^ new data is required for >1 kGy exposures so optimum absorbed doses can be discovered for either acetaldehyde or methanol conversion. Additionally, considering a green chemistry metric, such as mass productivity, is an essential context for any radiolytic process implemented industrially,^22^ especially radiolytic processes that are limited by the rate of energy input, which dictates catalysis and reaction kinetics.

Previous studies on ethylene glycol have reported a radical chain rearrangement reaction for acetaldehyde synthesis,^20,23−25^ using diluted samples (<6.2% wt %) and low doses (∼0.8 kGy), except for Barker’s report in which a 100 kGy dose was utilized. The studies that describe the rearrangement reaction report notable *G*-values greater than 18 μmol J^–1^ but neglect to consider resource conversion, resulting in low mass productivity values of ∼0.006%.^23^ Some works have claimed that acetaldehyde *G*-values can reach ∼20,700 μmol J^–1^ for low-dose, low-dose-rate conditions (1.6 kGy at 6.6 Gy min^–1^).^26^ However, these claims were based on indirect measurements of acetaldehyde, and these extraordinarily large *G*-values have not been reproduced. The literature on ethylene glycol radiolysis includes the report of the influence of temperature on methanol synthesis with *G*-values of 0.56 and 0.72 μmol J^–1^ at 0 and 60 °C, respectively.^21^ This temperature dependence for methanol production suggests the fragmentation of weak C–C bonds during primary physical reaction time scales (<10^–15^ s) which would be temperature dependent. It is predicted that this process would be promoted by higher-dose-rate irradiations and higher linear energy-transfer (LET) values, which are the average quantity of energy that is lost per unit path length as a charged particle travels through a medium. On the other hand, low-LET and low-dose-rate irradiation of concentrated samples would promote the acetaldehyde process.

**Figure 1:**
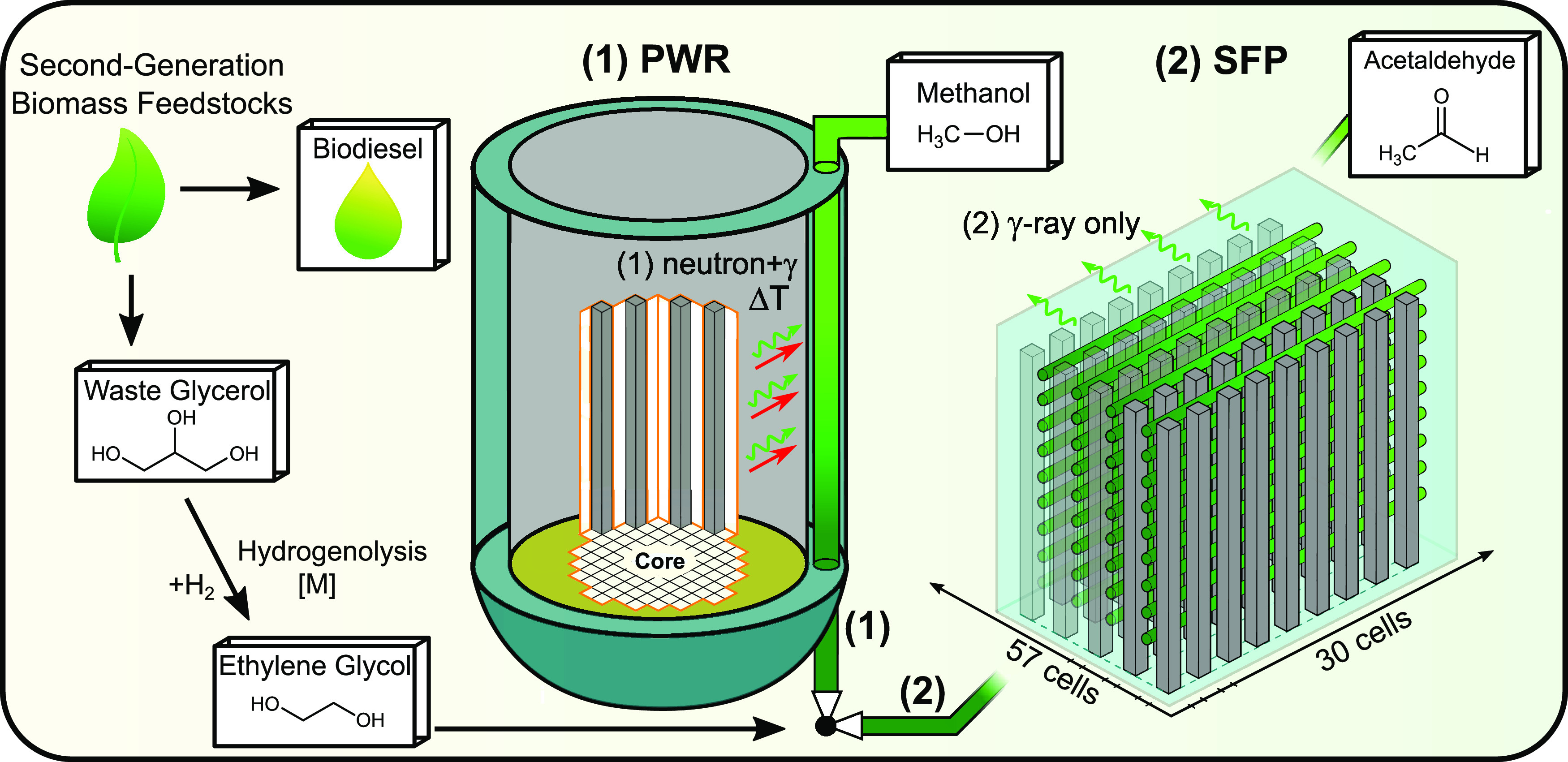
Schematic illustration of the nuclear biorefinery process. The two available options are (**1**) radiolytic methanol production through thermally assisted high-dose-rate, high-absorbed, high-LET, mixed-field irradiations from a PWR system or (**2**) acetaldehyde production with low-dose-rate, low-LET, γ-ray-only irradiations using a spent fuel pool (SFP) system. Additionally, utilizing γ rays from dry casks has also been identified as a feasible option.

A few reports have explored the irradiation of concentrated ethylene glycol samples for large, absorbed doses (∼100 kGy) that prioritize feedstock conversion. Additionally, few experimental studies explored high-fluence, high-LET (linear energy transfer), ionizing radiation from an active nuclear reactor for ethylene glycol radiolysis. The two main products of either acetaldehyde or methanol could be synthesized as desired, depending on the irradiation conditions. Figure 1 illustrates the flexibility of an ethylene glycol scheme using the multicomponent radiation fields from a nuclear facility to selectively produce methanol or acetaldehyde in two different systems. The pressurized water reactor (PWR) coproduction system (1) presents the option of irradiating organics with a mixed field (neutrons + γ rays) using radiation directly from the PWR to produce methanol selectively. System (2) offers the option of utilizing waste γ-ray irradiation from spent fuel cells in a spent fuel pool (SFP) production system to selectively produce acetaldehyde.

## Experimental Section

### Materials and Sample Preparation

The methods employed in this work are similar to those of a previously published paper by Plant et al.^27^ but adapted for the ethylene glycol precursor. Ethylene glycol (>99%) was purchased from Honeywell. All chemicals were used as supplied. To ensure a deaerated atmosphere, ethylene glycol samples to be irradiated were capped within an MBRAUN UNIlab Pro N_2_ nitrogen glovebox with atmospheric H_2_O and O_2_ concentrations of ∼0.7 ppm and <0.5 ppm, respectively. Butan-2-ol (99.9 mass %), purchased from Sigma-Aldrich, was used as an internal reference standard for the analyte calibration curves and added to samples after their irradiations. Chemical analytical standards of methanol (99.9%) and acetaldehyde (50 mass % in ethanol) were purchased from Sigma-Aldrich. Ethanol (99%), purchased from Fisher Scientific, was used for the dilution of the radiolytic samples before GC-MS analysis to lower sample viscosity. The liquid samples for GC analysis were prepared utilizing the gravimetric method with a Fisherbrand FB73651 mass balance with a stated accuracy (repeatability) of ±0.1 mg. The mass measurement errors are negligible when compared against the relative standard deviation (RSD) percentage for the calibration curves (between 5 and 20%) and absorbed dose uncertainty (10%) which contribute to *G*-value uncertainties. Polypropylene Argos Polarsafe cryovials (5 mL, external thread) were used as irradiation vessels, which were purchased from Fisher Scientific. The 5 mL vials were approximately filled with 1 mL of ethylene glycol inside the nitrogen glovebox and weighed using the Fisherbrand mass balance.

### Irradiations

Ethylene glycol samples were irradiated using the 250 kW TRIGA Mark II fission reactor at the Jožef Stefan Institute (JSI) as described in the literature.^28^ The 70 core fuel elements comprised 20% enriched ^235^U in a ZrH composite material. At a steady-state power of 250 kW, the maximum neutron and γ-ray fluence of 1.175 × 10^13^ and 1.21 × 10^13^ cm^–2^ s^–1^, respectively, is available from the triangular channel (TriC) of the reactor.^29^ All ethylene glycol samples were irradiated in the triangular irradiation channel of the TRIGA reactor with either only delayed γ rays during reactor shutdown or a mixed field (neutron + γ rays) with the reactor operating. During reactor shutdown, samples were irradiated with a γ-only dose at an average of 40 Gy min^–1^ from the activated nuclei in the fuel rods. During reactor operation, the samples were irradiated at a dose rate of between 1600 and 6500 Gy min^–1^ for mixed field irradiations. For the absorbed dose dependence study, ethylene glycol samples were irradiated with 20, 40, 50, 60, 80, or 100 kGy of absorbed dose with either irradiation mode (shutdown or operating). For the dose-rate-dependence study, samples were irradiated with a mixed-field dose rate of 520, 1310, 3270, or 8170 Gy min^–1^ for an absorbed dose of 50 kGy with the reactor in operational mode. For experimental dosimetry, two calculation methods were employed to determine the dose rates (in Gy s^–1^) for the two different irradiation modes in the triangular channel. First, the operational mode utilized the existing validated, MCNP TRIGA model, fluence-to-dose factors and the ENDF/B-VIL.0 nuclear library for the mixed-field dose rate.^28−31^ A substitute for a tissue analogue was used, ethylene glycol. A dose factor of 5.44 × 10^–4^ Gy s^–1^ W^1–^ was determined for the total mixed-field dose rate from the MCNP model. Second, for shutdown mode, dose rates were calculated based on the response of a calibrated ionization chamber together with the power reading of the reactor as measured in the linear channel.^32^ A factor of 14250 Gy s^–1^ W^1–^ was determined for γ-ray-irradiated samples in the triangular channel with an uncertainty of ∼10%. Groups of samples were irradiated for a specified time depending on the dose quality and desired absorbed doses.

### Chemical Analysis

All irradiated samples were analyzed within 30 days of their irradiation and 40 days of preparation. All samples (irradiated and control samples) were diluted volumetrically with ethanol in an ∼10:1 mass ratio and monitored via gravimetric measurements due to the high viscosity of ethylene glycol. Approximately 40 μL of a 1 mg mL^–1^ diluted stock solution of the internal standard, butan-2-ol (in ethanol), was added to each sample for the internal standard calibration methodology. The internal standard concentration of butanol in each of the diluted samples was separately calculated based on gravimetric measurements of the stock solution. Diluted samples were analyzed using a Shimadzu TQ8040 gas chromatography-mass spectrometer (GC-MS) equipped with an AOC 6000 autosampler. Shimadzu’s LabSolutions GC-MS software (v4.4) was used for data capture, analyte confirmation using analytical standards, and further quantitation analysis. The same software was used as an interface for a comparison between the measured fragmentation patterns and the NIST 11 MS standard reference database. The separations were performed using a 10-m column guard and a Zebron 624-Plus analytical column with a length of 30 m × 0.25 mm inner diameter and a film thickness of 1.4 μm. The injector temperature was set to 300 °C, and the oven program was set as follows: 40 °C (10 min); ramp of 25 °C min^–1^ to hold at 300 °C (2.6 min). Split injections were used with a volume of 1 μL, with a split ratio of 20:1 with a constant column flow of 1.71 mL min^–1^ during a run. The carrier gas used was helium with a purity of 99.999%. The detector and interface temperatures were set to 250 and 300 °C, respectively. The MS detector was set to full scan mode at a scan speed of 1000 Da s^–1^ between the mass–charge ratio (*m*/*z*) range of 30–300.

Due to the coelution of the secondary acetaldehyde and broad methanol peak, quantitation was achieved via the postprocessing of the mass–charge fragments of 31 *m*/*z* for methanol and 44 *m*/*z* for acetaldehyde. Figure S2 in the Supporting Information shows an example of the postprocessing of the fragments for a 100-kGy-irradiated ethylene glycol sample. The concentration of the radiolytic products within the diluted samples was measured directly using internal calibration curves and the concentration of the internal standard (butan-2-ol) in the diluted sample. Total product moles were calculated from the concentration by adjusting for the mass fragment extracted and the volumetric dilution ratio. The values for radiation chemical yields (*G*-values) were calculated using the moles of the analyte determined in the irradiated organic sample and divided by the energy into the same organic sample. The energy into the organic sample was calculated using the absorbed dose calculations and the starting mass of the organic sample before irradiation. Errors for the concentrations were derived from the relative standard deviation (RSD%) of the specific calibration curve used. The final error calculations for the radiation chemical yields were determined using RSD% of the initial analyte concentration, the uncertainty in volumetric and gravimetric dilutions, and the uncertainty for absorbed dose. Total uncertainty for *G*-value data points is in the range of ±(10–20%) depending on the sample, analyte, and calibration curve used.

### Particle Transport Simulations

Particle transport simulations were performed to determine dose rates in both irradiation scenarios of a typical PWR and a spent fuel pool. These simulations were achieved using the validated MCNP (Monte Carlo N-Particle Transport code (version 6.1.1))^33^ on one node of a 40-core (Intel Xeon Gold 6148) computer cluster. Each scenario introduced stainless-steel pipes that contained the ethylene glycol organic phase. The γ-ray ambient dose equivalent H*(10) in the organic phase was calculated using the flux-to-dose conversion factors from the ICRP-21 report^34^ and the JEFF-3.3 nuclear data library.^35^ Additionally, the neutron absorbed dose was calculated using the track length estimates of the volume average energy deposition (F6:n,p) tally type from the validated neutron fields of the PWR MCNP model.

### PWR Model

The typical PWR MCNP model based on the Krško PWR was developed at the JSI for determining dose fields throughout the containment building and calculating the expected detector responses in the biological shield surrounding the reactor pressure vessel. This PWR MCNP model has been validated via multiple experiments that can accurately determine γ-ray and neutron dose fields.^30,36^ Stainless-steel pipes (4 m height, 5 cm outer radius) were internally coated with indium layers of 2 or 4 mm to increase the total dose rate via additional γ rays via neutron capture reactions. The remainder of the stainless-steel pipe was filled with ethylene glycol. Table S5 gives the key values for each model used. The pipes were positioned in the reactor cavity between the pressure vessel and the biological shield. The simulations were performed for the case of an operating reactor, resulting in a mixed γ-ray and neutron field. Because of the large attenuation between the particle source and the pipes where the doses need to be calculated, a variance reduction of the particle transport simulation was needed. The ADVANTG code was used to prepare effective variance reduction parameters.^37^

### Spent Fuel Pool (SFP) Model

The spent nuclear fuel pool model was adapted from previous models with glycerol but with the organic being ethylene glycol.^27^ Initially, 10 fuel elements from the typical PWR model were modeled in a tank of borated water. The γ-ray source spectrum and activity were determined based on a typical burnup scenario (46274.21 MWd/tU). Only one stainless-steel pipe (2 m length, 4.8 cm inner radius, 5 cm outer radius) filled with glycerol at the middle height of the fuel elements (at 183 cm) was modeled. Based on the previous use of this model for glycerol, the dose rate was increased by a factor of 1.04 based on the γ-ray dose-rate differences for ethylene glycol from the previous PWR model.

### Scale-up Calculations

For the determination of the maximum yearly production capacity of each scenario, the mass productivity values as per Table S2 (for a specific dose) were combined with the values of the dose rate and the volume of the irradiated organic material from the MCNP models. The SFP model and the TRIGA reactor in shutdown mode are assumed to be comparable in terms of the *G*-values and mass productivity products for acetaldehyde and methanol due to similar γ-ray dose rates. The 5 × 2 matrix SFP model that carries the organic mixture was extended for ten 0.1 m × 12 m pipes in the vertical axis. The volume for irradiation was then expanded to the maximum operational capacity of 1710 spent fuel elements (30 × 57 matrix) in the pool, totaling 560 mixture-carrying pipes with a total irradiation volume of 5.28 × 10^7^ m^3^. The MCNP model for the PWR model was expanded for a maximum of 120 organic-carrying pipes within the cavity of the reactor vessel with a total organic irradiation volume of 3.19 × 10^6^ m^3^. Further parameters for the MCNP models are given in Table S5. For consistency with the empirical data, scaled-up volumes would be irradiated with either 100 or 20 kGy for the PWR or SFP system, respectively. For the yearly maximum production capacity of methanol and acetaldehyde for countries within the geographical area of Europe, the capacity is expanded relative to the nuclear electrical output of each country compared to the electrical output of the Krško PWR. It is assumed that other SFP facilities have similar maximum fuel cell capacities, total dose rates, and potential irradiation volumes as the Krško SFP facility. A similar extrapolation was conducted for all 422 worldwide operational reactors (as of April 19, 2023).

### Instrumental Neutron Activation Analysis (INAA)

The activated nuclei generated via neutron fields in ethylene glycol were characterized using the k0-instrumental neutron activation standard working procedure.^38,39^ 1.4 g amount of ethylene glycol was loaded into a polyethylene ampule and irradiated in the carousel facility of the TRIGA reactor. Samples were exposed to 270 kGy thermal neutrons and 250 kGy of γ rays. The γ-ray spectra of the samples were measured by using a high-purity germanium (HPGe) detector. The peak areas of specific and their related radionuclides were characterized using HyperLab 2002 software. The γ-ray spectra of the samples were measured at intervals of 0.5, 4, 11, and 22 days after irradiations.

## Results and Discussion

**Figure 2:**
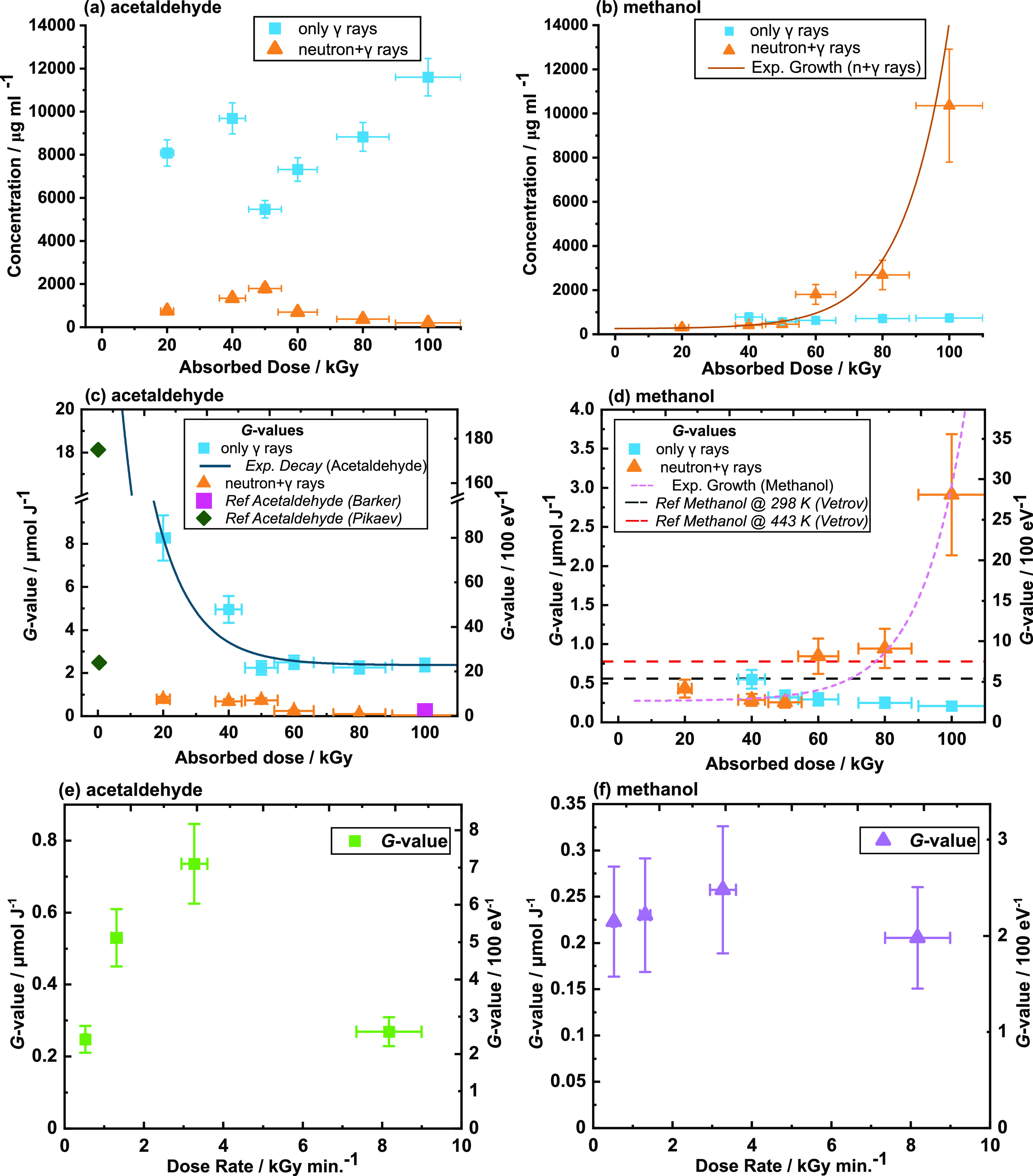
Concentrations and radiation chemical yields (*G*-values) of acetaldehyde and methanol from irradiation with neat ethylene glycol, for either specified dose of either γ-ray (cyan) or neutron + γ-ray (orange) irradiations. Concentrations of acetaldehyde and methanol are given in panels (a) and (b), respectively, for both irradiation modes. Samples in panels (a)–(d) were irradiated with dose rates between 18 and 40 Gy min^–1^ for γ rays and between 1600 and 6500 Gy min^–1^ for mixed-field irradiations. Corresponding *G*-values derived from the concentrations are displayed in panels (c) and (d). Additionally, *G*-values as a function of dose rate for (e) acetaldehyde and (f) methanol for 50 kGy neutron + γ-ray irradiation are given. *x*-axis error bars derive from absorbed dose uncertainties; *y*-axis error bars represent the combination of the relative standard deviation (RSD%) of the analyte concentration calibration curves and absorbed dose uncertainties for each sample. The complete data set is given in the Supporting Information, Tables S2 and S3. Statistics for the functions shown in panels (a) and (b) are given in Table S4. *Reference *G*-values from Barker irradiated 64.5 mM ethylene glycol in H_2_O,^23^ and Pikaev irradiated 1 mol dm^–3^ ethylene glycol solutions buffered with 0.1 M KOH that were deaerated or saturated with N_2_O gas.^20^ Vetrov fails to mention sample concentration or absorbed dose.^21^

Ethylene glycol was exposed to either two different types of irradiation: γ-only or a mixed-field comprising neutrons + γ rays, using a Mark II TRIGA reactor.^28^ Gas chromatography analysis with a mass spectrometer detector measured multiple stable radiolytic products (Supporting Information, Table S1 and Figure S1). Formaldehyde, acetaldehyde, methanol, ethyl acetate, acetic acid, 2-methyl dioxolane, 1,2-ethanediol, monoacetate, and diethylene glycol were all detected as products from low-dose-rate, γ-ray only exposures. However, high-dose-rate, mixed-field irradiations resulted in the detection of only formaldehyde, acetaldehyde, methanol, 2-methyl dioxolane, and diethylene glycol. Acetaldehyde and methanol were identified as the most reliable and consistent analytes, producing large peak areas suitable for quantitation and subsequent comparisons across both irradiation types and the absorbed dose range. Figure 2a,b displays the concentrations of acetaldehyde and methanol, respectively, detected in neat ethylene glycol samples for each irradiation quality type. Figure 2c,d displays the corresponding radiation chemical yields (*G*-values) of acetaldehyde and methanol, respectively, as a function of the absorbed dose from each irradiation quality type. In addition, Figure 2e,f illustrates the *G*-values of acetaldehyde and methanol as a function of the dose rate for 50 kGy mixed-field irradiations.

Figure 2a shows that the concentration of acetaldehyde for γ-ray exposures increases with absorbed dose which is the expected trend for radiolytic product generation.^40^ A similar trend is seen for mixed-field exposures up to 60 kGy but concentrations then decrease, indicating the decomposition of acetaldehyde or preferential generation of other products. Figure 2b shows that the concentration of methanol remains steady at ∼700 μg mL^–1^ above 20 kGy for γ rays. However, methanol concentration grows exponentially with increased mixed-field doses. More recent radiolysis literature has suggested the reporting of dose constants (in kGy^–1^) to evaluate the dose requirements for process radiolysis systems.^41^ Logarithmic plots of analyte concentrations are given in the Supporting Information, Figure S4, and corresponding values are listed in Table S4. The dose constant for acetaldehyde production via γ-ray exposure (asymptotic exponential) was determined to be 0.00214 kGy^–1^. The dose constant for methanol production via the mixed-field neutron + γ-rays (exponential growth) was determined to be 0.0193 kGy^–1^. A literature dose constant value for acetaldehyde production of 2.298 kGy^–1^ was calculated from γ rays,^20^ with concentration data collected at a significantly lower dose range between 0.0125 and 0.1625 kGy.

Figure 2c shows that acetaldehyde *G*-values exhibit a nonlinear relationship with increased absorbed dose under only γ-ray irradiation. Acetaldehyde shows a significant drop in *G*-value from its highest point at 8.28 ± 1.05 μmol J^–1^ at 20 kGy and 2.37 ± 0.30 μmol J^–1^ at 100 kGy. This suggests higher *G*-values at lower doses, which is consistent with the previous study by Pikaev.^20^ Notably, the decreases in acetaldehyde *G*-value (*y*) exhibit an exponential dependence with absorbed dose (*x*), as per, *y* = 2.2 + 30.4 e^*–x*/12.8^ μmol J^–1^. Additionally, acetaldehyde *G*-values at 100 kGy exceed those of the equivalent absorbed dose samples from the literature by Barker,^23^ which can be attributed to the more concentrated sample in this research. The *G*-values for acetaldehyde are comparatively lower for mixed-field irradiations than for γ-ray-only irradiations for the same absorbed dose, at 0.78 ± 0.12 μmol J^–1^ for 20 kGy. In Figure 2d, methanol *G*-values decrease with an increased γ-ray dose as expected. However, with an increasing mixed-field absorbed dose, methanol *G*-values rise significantly from 0.43 μmol J^–1^ at 20 kGy to 2.91 μmol J^–1^ at 100 kGy, corresponding to an exponential growth function of *y* = 0.27 + 0.006*e*^–(*x*–17.4)/13.4^ μmol J^–1^. This rise is accompanied by a drop in acetaldehyde *G*-value from 0.74 to 0.24 μmol J^–1^ from 50 to 60 kGy, respectively, when irradiating with neutron + γ rays indicating competing reactions between acetaldehyde and methanol for mixed-field irradiations.

Additionally, ethyl acetate and acetic acid were detected from γ-ray-only irradiations but exhibited low relative concentrations with *G*-values of 0.11 ± 0.02 and 0.20 ± 0.07 μmol J^–1^ for 50 kGy, respectively. The *G*-values of ethyl acetate and acetic acid did not significantly change with only γ-ray absorbed dose. Further data have been given in the Supporting Information, Table S2. The degradation of the polypropylene vials generating volatiles is thought to be negligible given the available high-dose studies reporting trace yields.^42−44^ Furthermore, repeating these exposures with borosilicate vials capped with aluminum–silicone septa shows comparable *G*-values of acetaldehyde and methanol to the polypropylene vials. This comparison is shown in Figure S3 in the Supporting Information, which shows the acetaldehyde *G*-values and mass productivity data points for both types of exposures and vials. Methanol is not listed as a polypropylene degradation product in the literature. However, acetic acid is reported as a polypropylene degradation product which may minorly interfere with the acetic acid concentrations measured from ethylene glycol.^42^

**Figure 3:**
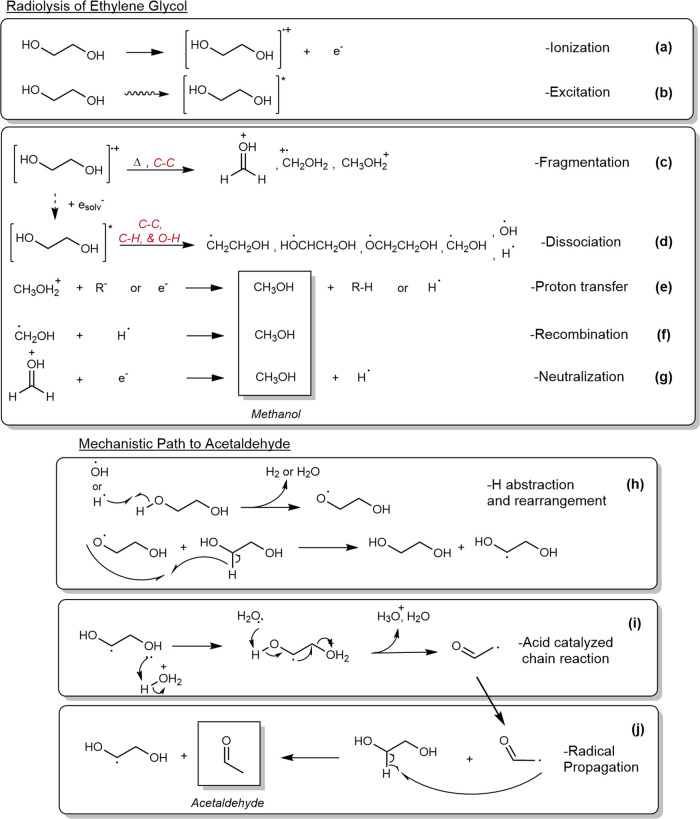
Physical and physicochemical mechanisms relevant to acetaldehyde and methanol synthesis from ethylene glycol radiolysis. (a, b) Ionization and excitation of ethylene glycol, respectively, (c) fragmentation of the ionized species,^48,49^ (d) dissociation of the excited species to radicals, (e) acid–base proton transfer to methanol, (f, g) other recombination and neutralization examples for methanol, respectively. (h) Mechanism for the alkoxyl (C–O^•^) radical and conversion to the more thermodynamically favorable hydroxy (^•^C–O) radical. (i, j) Removal of H_2_O for the hydroxy radical using an acidic species while reproducing the hydroxy radical in another molecule.^20,23,24^ The radical chain rearrangement reaction (h–j) was expanded upon from that reported in the literature. Reactions reproduced with permission from ref (20) where necessary.

The dose-rate dependence on acetaldehyde, as illustrated by Figure 2e, is not clear, although it was predicted that a higher *G*-value would be observed for lower dose-rate exposures of the mixed field (0.52 kGy min^–1^). A higher expected *G*-value was due to the previously reported chain rearrangement reaction that relies on spur diffusion-limited radical interactions, implying a lower volume of spur overlap, higher rates of diffusion, and hence more acetaldehyde. The proposed catalytic mechanisms illustrated in Figure 3 expand on previously reported mechanisms.^20,24^ In Figure 2f, the dose-rate dependence of the methanol *G*-values appears to be independent of the mixed-field dose rate, indicating that temperature could be the factor promoting C–C bond cleavage and increasing methanol generation, as discussed later.

The proposed reaction mechanisms for acetaldehyde and methanol production can be understood by relating them to the *G*-value data, relating these to the expected time scales of when specific reactions occur. This requires further definitions of the time-scale stages of a single radiolytic interaction. The physical stage of a radiolytic interaction occurs within 10^–15^ s of the initial ionization event, where energy is deposited in the medium as energetic volumes called spurs (<100 eV), blobs (<500 eV), or short tracks (<5000 eV).^45^ Within these energetic volumes, the molecular radical cations (M^+•^), excited molecular species (M*), and electrons are created initially.^46^ The physicochemical stage encompasses radical and ion reactions within 10^–15^ to 10^–12^ s of the ionization event. In the final chemical stage (10^–12^ to 10^–6^ s), diffusion kinetics become dominant. Here, diffusion-limited chemical reactions start to dominate as energetic volumes expand.^47^ Due to the large number of possible reactions associated with the numerous radicals and ions, only the main reactions and species pertinent to acetaldehyde and methanol production are described.

The main physical and physicochemical mechanisms of ethylene glycol radiolysis can be predicted by extrapolating information from electron ionization (EI) and density functional theory (DFT) studies of ethylene glycol fragmentations,^48,49^ as well as by reference to existing radiolytic studies.^20,23,24,50^ In the physical stages, irradiation can cause a bound electron to be ejected (ionization) at higher energies, which generates a molecular radical cation ([HOCH_2_CH_2_OH]^+•^) or excites a bound electron to produce an excited molecular species ([HOCH_2_CH_2_OH]*) at lower energies, as per Figure 3a,b, respectively. During the physicochemical stages, the ethylene glycol radical cation is thought to fragment into several species including CH_3_O^+^, CH_2_OH_2_^+•^, and CH_3_OH_2_^+^, based on the available literature,^48,49^ signifying the preferential cleavage of the C–C bond for the direct radiolysis of ethylene glycol molecules, as indicated by Figure 3c. Other ionization-derived fragmentations are negligible as evaluated from ethylene glycol’s electron ionization mass fragment pattern,^51^ but recombination with a previously ejected electron will produce an excited molecular species.

The cleavage of the C–H, O–H, and C–O bonds remains possible via dissociative relaxation reactions, ion–molecule interactions, and indirect radiolytic reactions from species such as H^•^ and ^•^OH, as indicated by the species generated in Figure 3d. The direct measurement of C_2_ products such as acetaldehyde with high *G*-values highlights the prominence of these indirect reactions.^20,23,24,52^ The fragmented ions and radicals can resolve to form methanol via proton transfer, neutralization, or recombination mechanisms,^53,54^ as per the examples in Figure 3e,g. However, it is encouraged that more refined DFT studies be conducted to confirm these fragmentations. Other neutralization reactions of ions occur to produce excited molecules, radicals, and molecular products such as H_2_ or H_2_O which start to occur within the chemical stages from radiolytic events. The dissociation or neutralization of other ions and excited species, such as those shown in Figure 3c,d, can resolve to form a variety of products such as formaldehyde, diethylene glycol, and 2-methyl-1,3-dioxolane.^23^ Focusing on the mechanistic path toward acetaldehyde, the generation of the oxygen-centered alkoxyl radical (C–O^•^) is preferred kinetically to the hydroxy (^•^C–O) radical as described in radiolytic studies of similar alcohols.^55,56^

However, the alkoxyl radical is converted rapidly to the more thermodynamically favorable hydroxy radical, as per Figure 3h. Here, the prominent hydroxy radical can undergo the acid-catalyzed chain rearrangement reaction to produce acetaldehyde, as indicated by Figure 3i,j. Despite the irradiation of neat ethylene glycol samples in this work, the indirect effects continue to dominate for γ-only irradiated samples as indicated by superior acetaldehyde production as opposed to methanol production. The high acetaldehyde *G*-values at higher alcohol solute concentrations compared with the literature suggest a couple of conclusions: The H^•^ and ^•^OH radicals from alcohols are still produced in abundance via C–H and O–H cleavage, respectively, compared with the H^•^ and ^•^OH radicals from H_2_O radiolysis. Additionally, high acetaldehyde *G*-values suggest fast kinetics of the rearrangement reaction for acetaldehyde production, as opposed to recombination reactions. Based on similar work with diluting glycerol,^27^ acetaldehyde *G*-values could be boosted with a small dilution with H_2_O due to viscosity and diffusion effects. For mixed-field irradiations, since methanol *G*-values are shown to be independent of dose rate, the superior methanol *G*-values are thought to be linked to the increased likelihood of C–C bond fragmentations from increased sample temperatures caused by the higher cumulative absorbed doses and higher PWR core temperatures during irradiations.

In this research, the organic samples exposed to 100 kGy of mixed fields are within the triangular channel (TriC) of the JSI reactor at 200 kW for a duration of 918 s. In other reports, the TRIGA Mark II reactors can reach steady-state core fuel temperatures of 146 °C at 200 kW at equivalent positions to the TriC in the JSI reactor,^57^ suggesting radiation-assisted pyrolysis processes may be possible, as indicated by this work. Additional and conflicting reactions occurring within the physicochemical and chemical stages are given in the Supporting Information, Figure S5.

**Figure 4:**
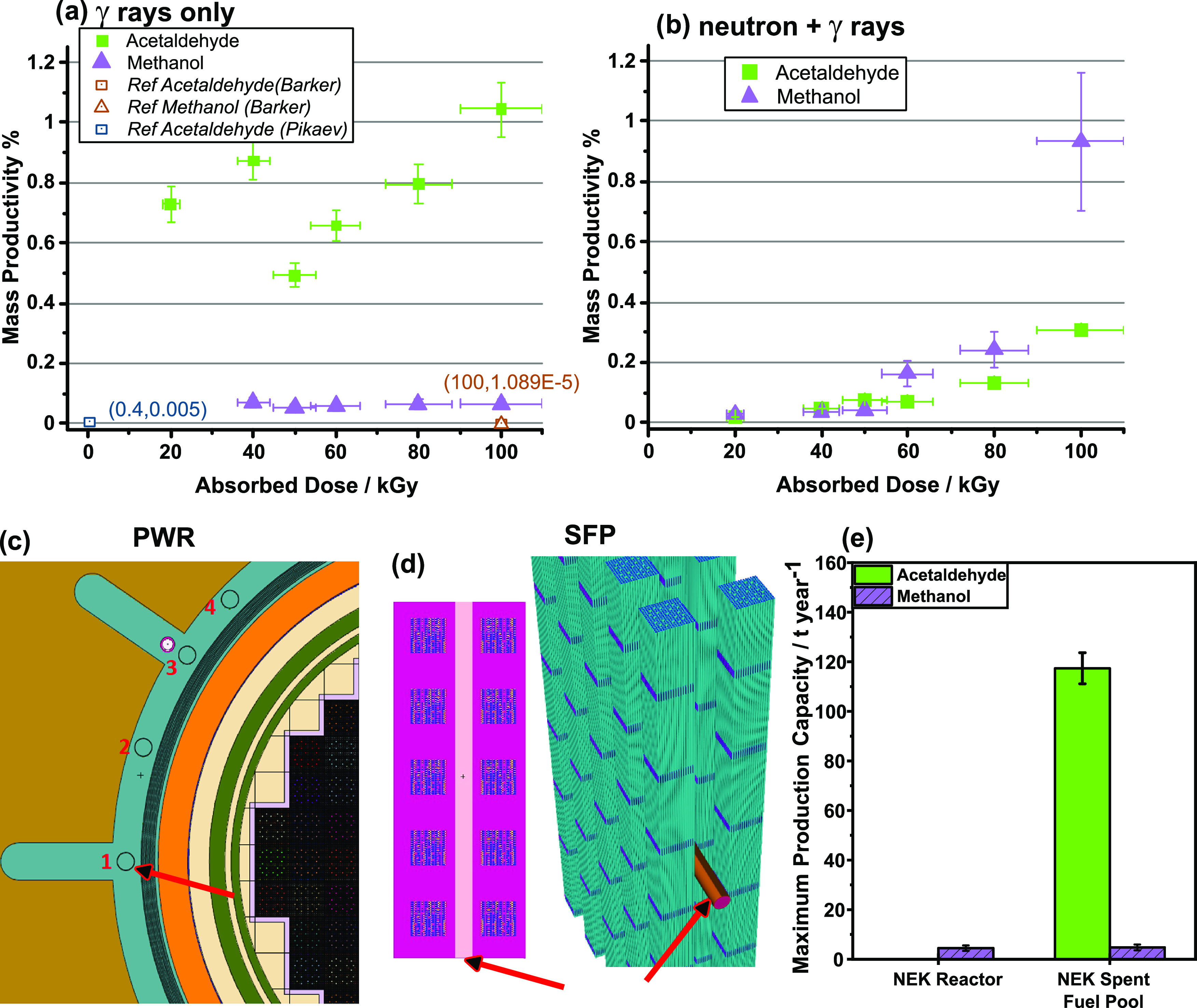
Mass productivity of acetaldehyde and methanol as a function of absorbed dose for (a) γ-ray only and (b) mixed-field neutron + γ-ray irradiations. (c) Two-dimensional (2D) cross-sectional diagram of the MCNP geometry depicting the 688 GW(e) Krško reactor and the organic-carrying pipes (black circles), (d) 2D and three-dimensional (3D) renders of the MCNP 2 × 5 matrix of spent fuel cells (blue) with horizontal organic-carrying pipes (red arrows), and (e) maximum production capacity values of acetaldehyde and methanol from the two systems.

Figure 4a,b illustrates the acetaldehyde and methanol mass productivity dependence on the absorbed dose, respectively, for either γ-ray only or mixed-field irradiations with the TRIGA reactor. It is anticipated that the mass productivity of acetaldehyde saturates and is consistent with an asymptotic regression function with absorbed dose due to conflicting reactions. The predicted steady-state equilibrium would exist where the rate of acetaldehyde formation matches its rate of reduction by the solvated electron species (as per Figure S5a) and chemical reactions with other compounds (as per Figure S5b–f).^50^ The mass productivity trend of methanol demonstrates independence to the γ-ray absorbed dose, suggesting a steady-state equilibrium has been reached by the 20 kGy γ-only exposures.

Figure 4b indicates that the mass productivity of methanol increases exponentially with a mixed-field absorbed dose, which can be attributed to temperature-assisted radiolytic fragmentation. Previous studies have shown that a minimum temperature of ∼500 °C is required for the conventional pyrolysis (thermal decomposition) of ethylene glycol to form an unselective array of molecules such as ethanol, acetaldehyde, ethane, and methane.^58^ The comparison between Figure 4a,b highlights that the mixed-field irradiations favor methanol production, suggesting a more selective cogeneration process that harnesses both heat and the underutilized ionization radiation from a PWR in a single catalytic process.

Figure 4c illustrates a 2D representation of a Monte Carlo particle transport code (MCNP) model of the 688 GW(e) Krško fission reactor, featuring organic-carrying pipes positioned vertically through the wall of the containment vessel at four different positions. Under normal operation, it is hypothesized that the organic-carrying pipes would be exposed to mixed-field radiation at elevated temperatures of ∼80 °C. Using the MCNP model, a total maximum achievable dose rate of 1.25 kGy hr^–1^ was calculated, with 79% derived from γ rays and 21% from neutrons. Figure 4d presents a 3D representation of the 2 × 5 matrix of spent fission fuel cells which emit only γ rays at 0.628 kGy hr^–1^ into a horizontal organic-carrying pipe for prioritizing acetaldehyde production. This model was expanded to a 30 × 57 matrix of cells for a maximum total of 1710 cells and 560 pipes, per the capacity of the spent fuel pool utilized by the Krško PWR.^59^Figure 4e illustrates the maximum production capacity for both acetaldehyde and methanol using the two different modes of irradiation. The maximum production capacity is dependent on the dose rate, mass productivity, and maximum volume of irradiation at a specified *G*-value and absorbed dose, with full model parameters provided in Table S5. Further renderings of the MCNP models are given in Figure S6.

The PWR model demonstrates that the selective production of methanol over acetaldehyde yields an estimated annual production of 4.47 ± 1.10 t yr^–1^. However, the total production capacity of methanol remains comparable to the γ-only SFP model, where 4.76 ± 1.18 t yr^–1^ methanol is produced alongside the desired production of 117.4 ± 6.25 t yr^–1^ of acetaldehyde. For the scale-up scenarios, several factors control the maximum production capacity, including the *G*-value, dose rate, and irradiation volume. Here, the high-LET irradiation model is only twice the dose rate of the low-LET SFP model (1250 to 628 Gy hr^–1^) which, combined with the SFP model having a significantly larger irradiation volume, explains the lackluster capacity of the scaled-up PWR model. However, it is predicted that increasing the organic temperature within the PWR scenario would increase the achievable *G*-values and production capacities for methanol synthesis. New, generation-IV reactor designs could be constructed with chemical coproduction in mind to achieve higher dose rates, higher temperatures, and consequently higher mass productivities of methanol.

### Industrial Scale-up Network

We have extrapolated the production model for a single system described in Figure 1 to a network consisting of 170 operating nuclear power plants (NPPs) within a European geographical area (and their theoretically equivalent SFP sites). This extrapolation, relative to the nuclear electrical output of each country in Europe, is compared against Slovenia’s Krsko NPP presented in Figure S7.^60^ Furthermore, the production capacity of acetaldehyde or methanol is expanded to all 422 operating reactors worldwide, as listed in Table 1.

**Table 1:** Scale-up of the Various Models across a Possible Network of Equivalent Nuclear Sites in Geographical Europe (Proportional to the Total Power Output)^a^

	maximum production capacities, ×10^3^ t yr^–1^			
	(1) PWR cavity (neut. + γ)		(2) SFP system (γ-ray only)	
region	methanol	acetaldehyde	methanol	acetaldehyde
Europe (170 reactors)	0.99 ± 0.24	0.02 ± 0.00	1.05 ± 0.26	25.9 ± 1.4
World (422 reactors)	2.45 ± 0.61	0.04 ± 0.00	2.61 ± 0.65	64.5 ± 3.4
% of worldwide production capacity	0.002	0.003	0.002	4.962

a Data on nuclear power were accessed on April 19, 2023.^60^

At a maximum production capacity of 6.5 × 10^4^ t yr^–1^ of acetaldehyde worldwide, the best case for a spent fuel system would contribute to only 4.96% of the acetaldehyde supply worldwide (1.3 × 10^6^ t yr^–1^). Due to the large worldwide production of methanol, the radiolytic PWR cavity system would only contribute to 2.5 × 10^4^ t yr^–1^ or 0.002% of worldwide supply, confirming the PWR cavity system would have an insignificant real-life impact. Consequently, the production capacity advantages of the spent fuel pool production system are more significant compared to the PWR cavity system. Since methanol and acetaldehyde are produced simultaneously for either method, the separation of their azeotropic mixture could be industrially achieved through pressure swing distillation (PSD).^61^ While only ∼5% of worldwide acetaldehyde would be produced from a considerable number of radiolytic SFP systems (∼422), the optimization and improvement of this process could make this worthwhile in the future. Different radiolytic reactions may also be considered for future processes, especially if they display favorable *G*-values, such as bromination reported in legacy research.^62^ Along with our previous report,^27^ this research highlights the untapped potential of the associated γ-ray emission from spent fuel assemblies stored in pools to be used for radiation-induced, catalytic transformations. However, when utilizing neutronic-based fields, induced radioactivity in the product stream remains a valid concern as previously discussed in the literature.^52^ However, little radioactivity is produced if the material is a pure organic material, as elemental impurities present the most likely source of γ-ray-producing radioactive nuclei. To show this, supporting instrumental neutron activation analysis (INAA) of the irradiated ethylene glycol starting material was conducted for quantifying generated radioactive nuclei. As shown in the Supporting Information, Table S6, only bromine-82 and sodium-24 were significantly γ-ray active directly after 520 kGy mixed-field irradiations with a cumulative specific activity of 6880 Bq g^–1^ but this decreased to 10 Bq g^–1^ after 10 days due to short half-lives.

### Future Cogeneration Systems

While the cogeneration systems proposed in this work would require some significant changes to existing NPP reactors and spent fuel pool arrangements, designs for the Gen-IV VHTR (very-high temperature reactor) already incorporate secondary thermochemical cogeneration loops intended for hydrogen gas production.^5^ Four out of the six research Gen-IV reactors designated for hydrogen gas cogeneration by the IAEA highlight the focus of the industry toward thermochemical nuclear cogeneration.^63^ Here, the benefits of cogenerating hydrogen gas are noticeable at periods of high electricity supply and low prices (low demand, midday), with hydrogen generated and sold at a relatively higher price, avoiding the need for load-following power generation which is known to reduce reactor lifetimes.^64^

The cogeneration of hydrogen would improve the flexibility of NPP operations and would likely impact the value-adjusted-levelized cost of energy (VALCOE) positively.^65^ However, nuclear-derived “pink” hydrogen is only slightly more expensive at 159 $ MWh^–1^ in 2023 when compared against the minimum estimate of unsubsidized nuclear electricity of 141 $ MWh^–1^.^66^ This hydrogen value is extrapolated for the high-case, 20 MW electrolyzers which is the largest planned in the EU while utilizing the lower heating value (LHV) of 33.3 kWh kg^–1^ for hydrogen. While pink hydrogen compares well against green hydrogen (which sits at 221 $ MWh^–1^),^66^ the comparative value to electricity makes hydrogen appear insufficient to provide significant economic advantages. Without significant subsidies, “pink” hydrogen may only benefit nuclear prospects incrementally and does not remove the significant economic drawbacks of nuclear energy investment (i.e., high capital costs and low-value main product).

A more ambitious focus would be to pursue the cogeneration of higher-value products, such as commodity chemicals. While the cogeneration of chemicals would depend on the compound demands and nuclear regulations, it would present a more lucrative cogeneration proposition that would promote nuclear sector investment, would contribute toward net zero carbon targets, and could lead to sustainable reaction schemes targeting nonpetrochemical-derived sources of chemicals. Additionally, commercial VHTR designs capable of H_2_ cogeneration are not anticipated to be fully deployed until 2040,^67^ despite optimiztic claims by 2030 as per the World Nuclear Association.^63^ Admittedly with some modifications, the advantage of the SFP process presented in this work is that it would be in operation significantly sooner than future cogenerating commercial Gen-IV reactors.

## Conclusions

In summary, this study presents a chemical process for the selective synthesis of acetaldehyde or methanol from ethylene glycol using two distinct irradiation scenarios. The results demonstrate *G*-values of 8.28 and ∼0.55 μmol J^–1^ for acetaldehyde and methanol, respectively, with 20 kGy of low-LET γ-ray irradiations, which aligns with absorbed dose dependencies observed in the prior radiolysis art. By contrast, high-LET, mixed-field irradiations produced methanol at *G*-values of 2.91 μmol J^–1^ at 100 kGy, with acetaldehyde *G*-values at a lower value of 0.4 μmol J^–1^. A dose constant of 0.00214 kGy^–1^ was determined for γ-ray-generated acetaldehyde between 20 and 100 kGy, which is lower than the corresponding literature as larger absorbed doses are explored in this study.

This research presents realistic resource conversion values (mass productivity) for high-dose (∼20 to 100 kGy) radiolytic processes on neat reagents which the prior radiolysis literature has rarely explored. Maximum mass productivities for acetaldehyde and methanol were 1.045% and 0.933%, respectively, for the preferential irradiation modes and doses. This study also provides a temperature-assisted radiolysis C–C fragmentation mechanism for methanol formation using a high-LET, high-dose-rate, large-absorbed dose, and mixed-field exposures. The work expands on the acid-catalyzed chain rearrangement to acetaldehyde reported previously.

The maximum production capacities presented for the two scenarios demonstrate the greater appeal of the spent fuel pool system for acetaldehyde production, which could produce 117.4 t yr^–1^ per system and a theoretical 64.5 kt yr^–1^ worldwide, assuming that all 422 systems are in operation. This spent fuel pool production system utilizes otherwise wasted material (i.e., spent fuel assemblies) and their γ-ray emissions. Further research into these unconventional nuclear cogeneration processes, focused on high-value coproducts, could yield a better internal return rate on investment than hydrogen gas for Gen-IV cogeneration designs. Developing these industrially orientated, radiation chemical processes could improve the financial appeal of nuclear power, thus providing low-carbon electricity and supplying petrochemical-free, renewable chemicals.

## Data Availability

The data generated during this study are included in the published article and within the Supporting Information document. The MCNP code for particle transport is available via the Radiation Safety Information Computational Center (RSICC). The input for the MCNP code can be made available from the corresponding authors upon a reasonable request.

## Supporting Information Available

Contains tabulated concentration data, radiation chemical yields, parameters for function-specific trends, INAA data and supporting figures (PDF)
