# Supplementary material for: Nuclear Cogeneration of Methanol and Acetaldehyde from Ethylene Glycol Using Ionizing Radiation
Source: Ind Eng Chem Res. 2023 Dec 4;62(49):21152–63. doi: 10.1021/acs.iecr.3c03317 (PMC10722510; doi:10.1021/acs.iecr.3c03317)
Supplement: Supplementary file 1 — ie3c03317_si_001.pdf [file ie3c03317_si_001.pdf]

# *Supporting Information*

## Nuclear Cogeneration of Methanol and Acetaldehyde from Ethylene Glycol using Ionizing Radiation

*Arran George Plant†*, Bor Kos‡, Anže Jazbec‡, Luka Snoj‡, Malcolm John Joyce†, Vesna Najdanovic-Visak§*

†School of Engineering, Lancaster University, Lancaster, UK.
‡Jožef Stefan Institute, Ljubljana, Slovenia.
§Chemical Engineering and Applied Chemistry (CEAC), Energy & Bioproducts Research Institute (EBRI), Aston University, Birmingham, UK.

Corresponding author: Email: arran.plant@gmail.com

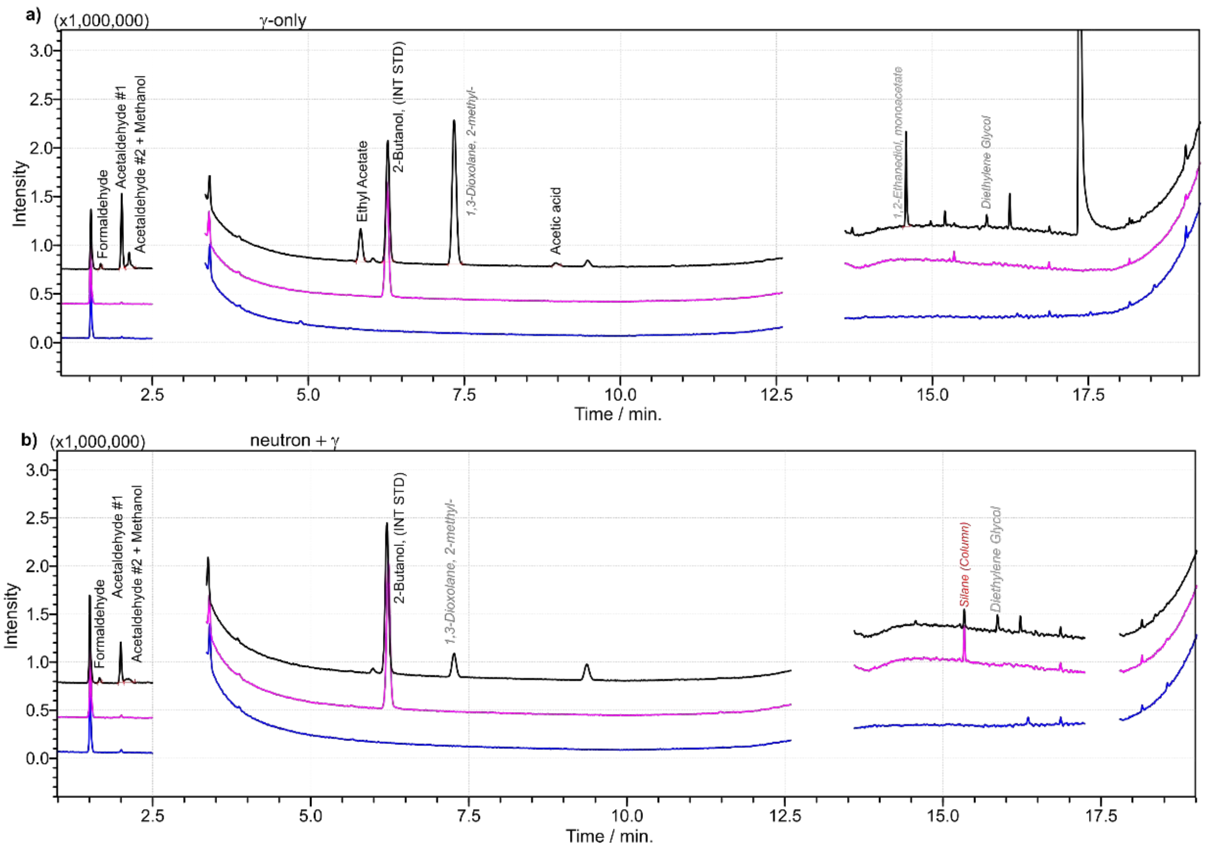


**Figure S1.** Total Ion Chromatogram (TIC) of irradiated ethylene glycol for 100 kGy of either a) γ-ray only or b) mixed-field neutron + γ-ray exposure.

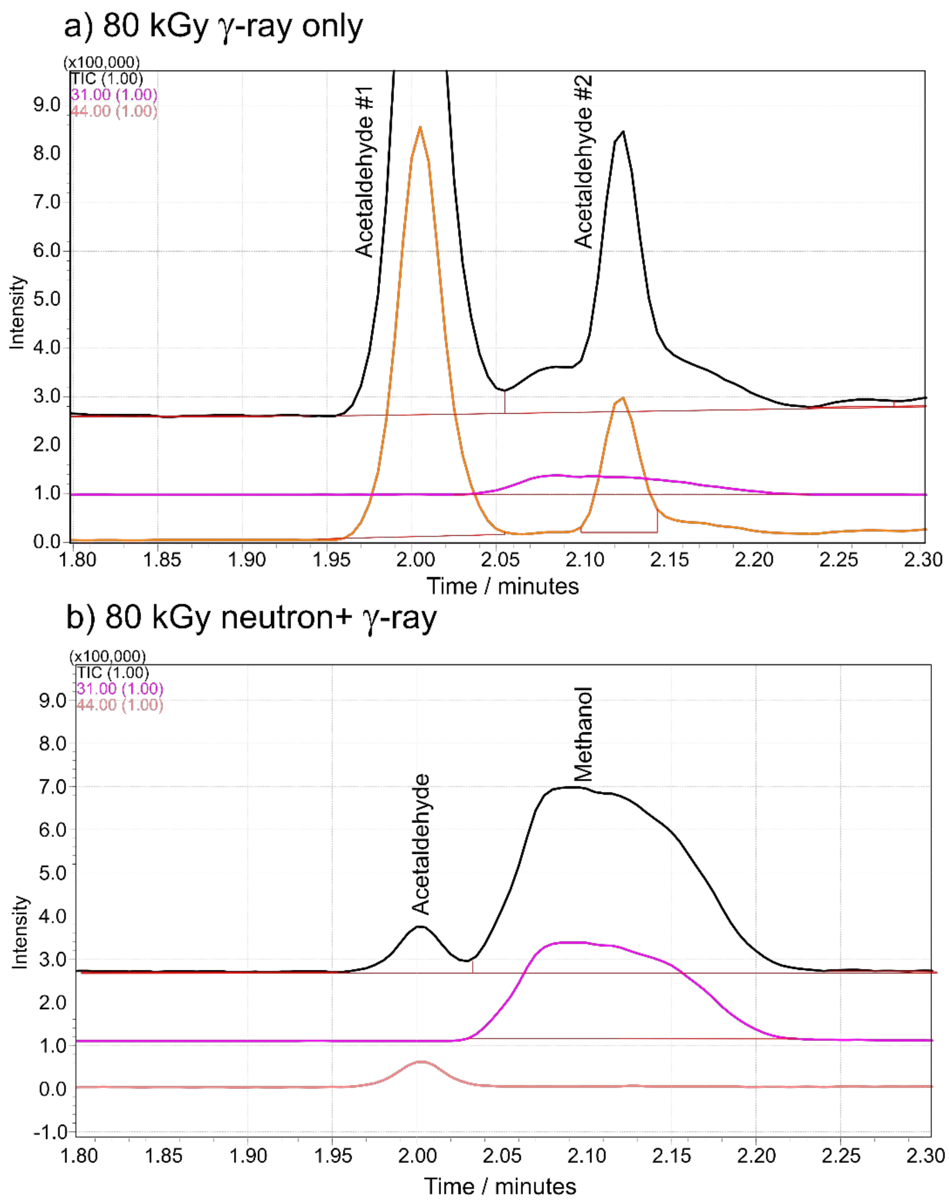


**Figure S2.** Post-processing of methanol (44 m/z, pink) and acetaldehyde (31 m/z, orange) fragment peaks from either a) γ-ray only exposure and b) neutron + γ-ray exposure.

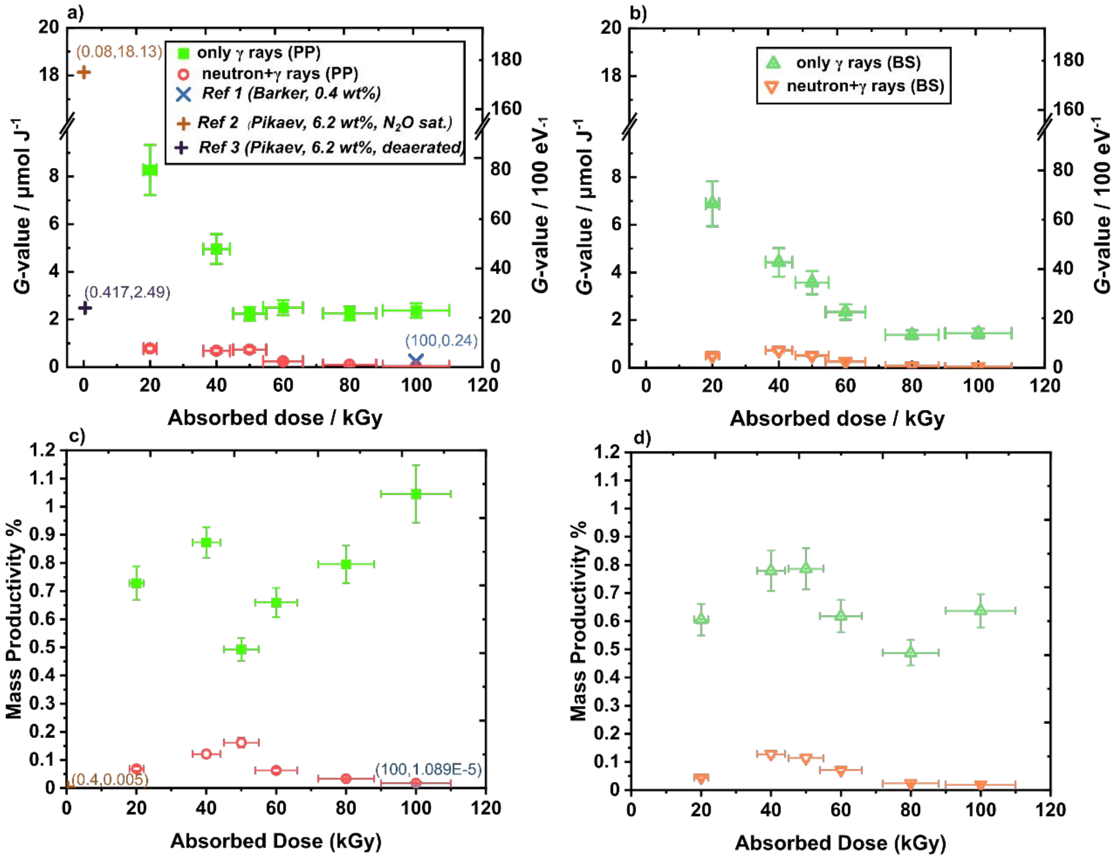


**Figure S3.** Radiation chemical yields (*G*-values) of acetaldehyde from irradiated ethylene glycol samples generated from either γ-ray only (green) or mixed-field neutron + γ-ray (red) irradiations. GC-MS measured concentrations from **a**) Polypropylene (PP) Argos Vials (green squares and red circles) and **b**) Borosilicate (BS) vials capped with aluminium/silicone septa (green or red triangles). Mass productivity (proportional to concentration) of acetaldehyde from **c**) (PP) 5ml Polypropylene Argos Vials and **d**) (BS) borosilicate vials.

**Table S1.** Mixed-field neutron + γ-ray or only γ-ray radiolysis of ethylene glycol: qualitative products detected using liquid sampling GC-MS techniques. Displayed products are for large-absorbed doses (>20 kGy). Molecule similarity comparison against the NIST11 MS spectrometry database, products were confirmed with analytical standards where applicable.

| Number | Product | Peak Retention Time (min.) | % Similarity | Confirmed Using Standards (Y/N) | Quoted in Literature of Ethylene Glycol or Similar Alcohols (Y/N)? |
|---|---|---|---|---|---|
| 1 | Formaldehyde | 1.67 | 97 | Y | Y |
| 2 | Acetaldehyde | 2.10 & 2.15 | 98 | Y | Y |
| 3 | Methanol | 2.15 | 98 | Y | Y |
| 4 | Ethyl Acetate | 5.80 | 98 | Y | N |
| 5 | Acetaldehyde, hydroxy- | 5.95 | 97 | N | Y |
| 6 | 1,3-Dioxolane, 2-methyl- | 7.29 | 97 | N | Y |
| 7 | Acetic Acid | 8.88 | 98 | Y | Y |
| 8 | 1,2-Ethanediol, monoacetate | 14.57 | 96 | N | N |
| 9 | Diethylene Glycol | 15.80 | 94 | N | Y |
| 10-24 | 14 Unidentifiable Products* | - | <88 | - | - |

*Suggested products unlikely to be accurate to low % similarity. The high retention time peaks are likely to be polymeric compounds with diverse arrangement of monomers.

**Table S2.** Absorbed dose dependence on concentrations, radiation chemical yields (*G*-values), and mass productivities of methanol, acetaldehyde, ethyl acetate and acetic acid from irradiated neat ethylene glycol samples (Figures 2 a and b).

| | **Dose Type (reactor mode)** | **only γ rays (Shutdown)** | | | | | | **Control** | **Mixed-field neutron + γ rays (Operational)** | | | | | |
|---|---|---|---|---|---|---|---|---|---|---|---|---|---|---|
| | **Absorbed Dose (kGy)** | 20 | 40 | 50 | 60 | 80.2 | 100 | NA | 20 | 40 | 50.4 | 60 | 80.2 | 100 |
| **Methanol** | **Concentration in neat Irradiated Sample (μg ml$^{-1}$)** | <LOQ* | 780 | 572 | 629 | 713 | 738 | <LOD* | 306 | 409 | 458 | 1808 | 2686 | 10356 |
| | ***G*-value (μmol J$^{-1}$)** | <LOQ | 0.548 | 0.322 | 0.295 | 0.251 | 0.207 | <LOD | 0.430 | 0.287 | 0.257 | 0.847 | 0.944 | 2.912 |
| | ***Mass Productivity %*** | <LOQ | 0.070 | 0.052 | 0.057 | 0.064 | 0.066 | <LOD | 0.028 | 0.037 | 0.041 | 0.163 | 0.242 | 0.933 |
| **Acetaldehyde** | **Concentration in Irradiated Sample (μg ml$^{-1}$)** | 8085 | 9688 | 5470 | 7316 | 8830 | 11599 | <LOD | 761 | 1345 | 1796 | 696 | 374 | 197 |
| | ***G*-value (μmol J$^{-1}$)** | 8.277 | 4.959 | 2.240 | 2.497 | 2.260 | 2.375 | <LOD | 0.779 | 0.689 | 0.735 | 0.238 | 0.096 | 0.040 |
| | ***Mass Productivity %*** | 0.728 | 0.873 | 0.493 | 0.659 | 0.796 | 1.045 | <LOD | 0.069 | 0.121 | 0.162 | 0.063 | 0.034 | 0.018 |
| **Ethyl Acetate** | **Concentration in Irradiated Sample (μg ml$^{-1}$)** | 114 | 210 | 516 | 713 | 494 | 701 | <LOD | <LOD | <LOD | <LOD | <LOD | <LOD | <LOD |
| | ***G*-value (μmol J$^{-1}$)** | 0.058 | 0.054 | 0.106 | 0.122 | 0.063 | 0.072 | <LOD | <LOD | <LOD | <LOD | <LOD | <LOD | <LOD |
| | ***Mass Productivity %*** | 0.010 | 0.019 | 0.046 | 0.064 | 0.045 | 0.063 | <LOD | <LOD | <LOD | <LOD | <LOD | <LOD | <LOD |
| **Acetic Acid** | **Concentration in Irradiated Sample (μg ml$^{-1}$)** | <LOQ | <LOQ | 662 | 678 | 649 | 850 | <LOD | <LOD | <LOD | <LOD | <LOD | <LOD | <LOD |
| | ***G*-value (μmol J$^{-1}$)** | <LOQ | <LOQ | 0.199 | 0.170 | 0.122 | 0.127 | <LOD | <LOD | <LOD | <LOD | <LOD | <LOD | <LOD |
| | ***Mass Productivity %*** | <LOQ | <LOQ | 0.060 | 0.061 | 0.058 | 0.077 | <LOD | <LOD | <LOD | <LOD | <LOD | <LOD | <LOD |

***<LOD= Below the limit of detection**

****<LOQ=Below the limit of quantitation**

**Table S3.** Dose rate dependence on G-values of acetaldehyde and methanol from neat ethylene glycol samples (Figure 2c and d). All samples were irradiated with 50 kGy of mixed field neutron + γ-ray irradiation.

| | **Dose Type (reactor mode)** | **Mixed-field neutron + γ rays (Operational)** | | | |
|---|---|---|---|---|---|
| | **Reactor Power (kW)** | 16 | 40 | 100 | 250 |
| | **Absorbed Dose Rate (kGy min.$^{-1}$)** | 0.52 | 1.31 | 3.27 | 8.17 |
| **Acetaldehyde** | ***G*-value (µmol J$^{-1}$)** | 0.248 | 0.530 | 0.735 | 0.269 |
| **Methanol** | ***G*-value (µmol J$^{-1}$)** | 0.223 | 0.230 | 0.257 | 0.205 |

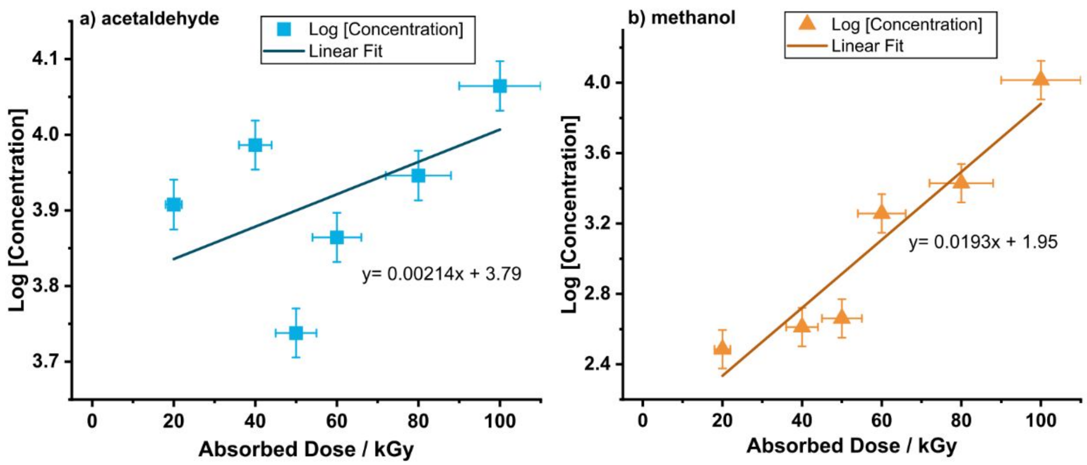


**Figure S4.** Absorbed dose vs log[concentration] plots for a) acetaldehyde (γ-ray only) and b) methanol (mixed-field). Where the dose constants, in $kGy^{-1}$ are indicated by the gradient value and listed further in Table S4.

**Table S4**. Parameters and statistics for plotted graphs the plotted absorbed dose vs Concentration, log[concentration], and *G* value relationships of acetaldehyde and methanol from neat ethylene glycol samples. (Plots shown in Figures 2b, c, d and S4). $\chi_\nu^2$ = Reduced Chi squared, $\chi^2$= Chi-squared, ν= degrees of freedom, n=number of data points in dataset.

| Product, Figure | Type | Irradiation Quality | *Equation* | $A_1$ | $C_0$ or $G_0$ (µg m$^{-1}$ or µmol J$^{-1}$) | $t_1$ | $x_0$ | $\chi_\nu^2$ ($\nu$,n) | $\chi^2$ |
|---|---|---|---|---|---|---|---|---|---|
| **Methanol, 2a** | **Concentration** | **Neutron+γ rays** | $C = C_0 + A_1 e^{(\frac{x-x_0}{t_1})}$ | 6.755 | 251.0 | 13.40 | *-2.22* | 1.5 (2,6) | 3.04 |
| **Acetaldehyde, 2c** | **G-value** | **Only γ rays** | $G = G_0 + A_1 e^{(\frac{-x}{t_1})}$ | 30.45 | 2.20 | 12.76 | *NA* | 2.9 (4, 6) | 11.92 |
| **Methanol, 2d** | **G-value** | **Neutron+γ rays** | $G = G_0 + A_1 e^{(\frac{x-x_0}{t_1})}$ | 0.006 | 0.27 | 13.44 | 17.45 | 3.5 (4,6) | 13.88 |
| | | | **For Dose Constants, kGy$^{-1}$** | | | | | | |
| **Product, Figure** | **Type** | **Irradiation Quality** | ***Equation*** | **m** (dose constant, kGy$^{-1}$) | **c** | | | $\chi_\nu^2$ ($\nu$,n) | $\chi^2$ |
| **Acetaldehyde, S4** | **Log [Conc.]** | **Only γ rays** | Log[C] = mx + c | 0.00214 | 3.79 | | | 10.6 (4,6) | 42.2 |
| **Methanol, S4** | **Log [Conc.]** | **Neutron+γ rays** | Log[C] = mx + c | 0.0193 | 1.95 | | | 6.7 (4,6) | 26.6 |
| ***Acetaldehyde (Pikaev, 1975)*** | **Log[Conc.]** | **γ rays** | Log[C] = mx + c | 2.298 | 1.298 | | | - | - |

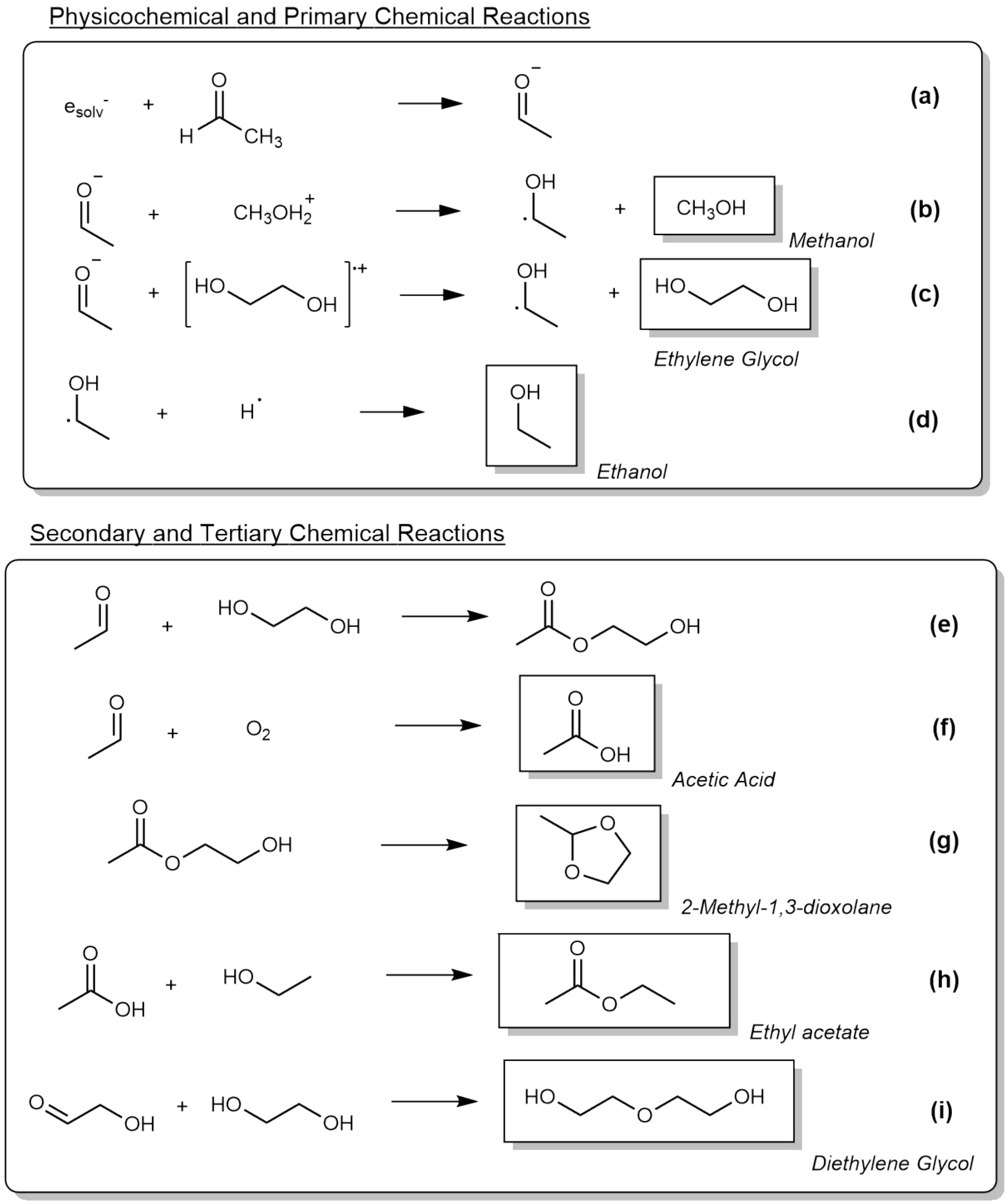


**Figure S5.** Extra physicochemical processes and other chemical reactions. These processes are extrapolated from the existing radiolysis literature.

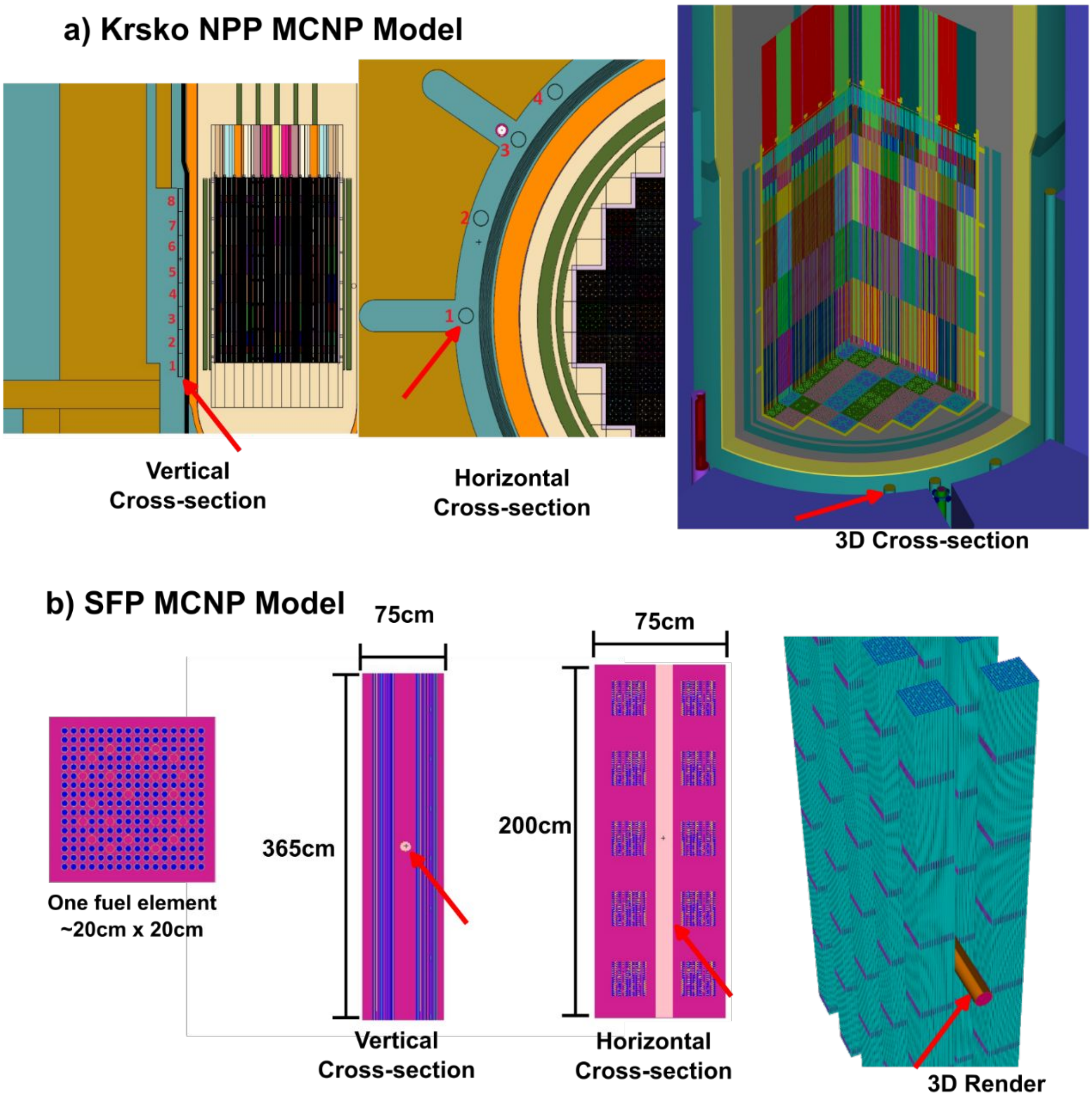


**Figure S6.** Further illustrations of the MCNP models for **a**) the NPP Krsko reactor, and **b**) the SFP system. Red arrows indicate the organic-carrying pipes.

**Table S5.** Parameters of the MCNP models explored in this work.

| Model | Organic-carrying Pipe Dimensions, (OD, L) m | Volume of pipe in model, $m^3$ | Total volume of scaled-up irradiation model, $m^3$ | Temperature, $^{o}C$ | Total Dose rate, kGy $hr^{-1}$ | Neutron Dose rate, kGy $hr^{-1}$ | Gamma Dose Rate, kGy $hr^{-1}$ | Absorbed Dose, kGy | Production Capacity Acetaldehyde, t $yr^{-1}$ | Production Capacity Methanol, t $yr^{-1}$ |
|---|---|---|---|---|---|---|---|---|---|---|
| PWR Krsko (cavity), No Indium Layer | 0.05, 4 | $2.99 \times 10^4$ | $3.47 \times 10^6$ | ~80 | 0.52 | 0.25 | 0.27 | 100 | - | - |
| PWR Krsko (cavity), 2 mm Indium Layer | 0.05, 4 | $2.66 \times 10^4$ | $3.19 \times 10^6$ | ~80 | 1.25 | 0.26 | 0.99 | 100 | 0.07 | 4.47 |
| PWR Krsko (cavity), 4 mm Indium Layer | 0.05, 4 | $2.43 \times 10^4$ | $2.92 \times 10^6$ | ~80 | 1.30 | 0.27 | 1.03 | 100 | - | - |
| Spent Fuel Pool | 0.1, 2 | $1.57 \times 10^4$ | $5.28 \times 10^7$ | 30 | 0.628 | - | 0.628 | 20 | 117.4 | 4.76 |

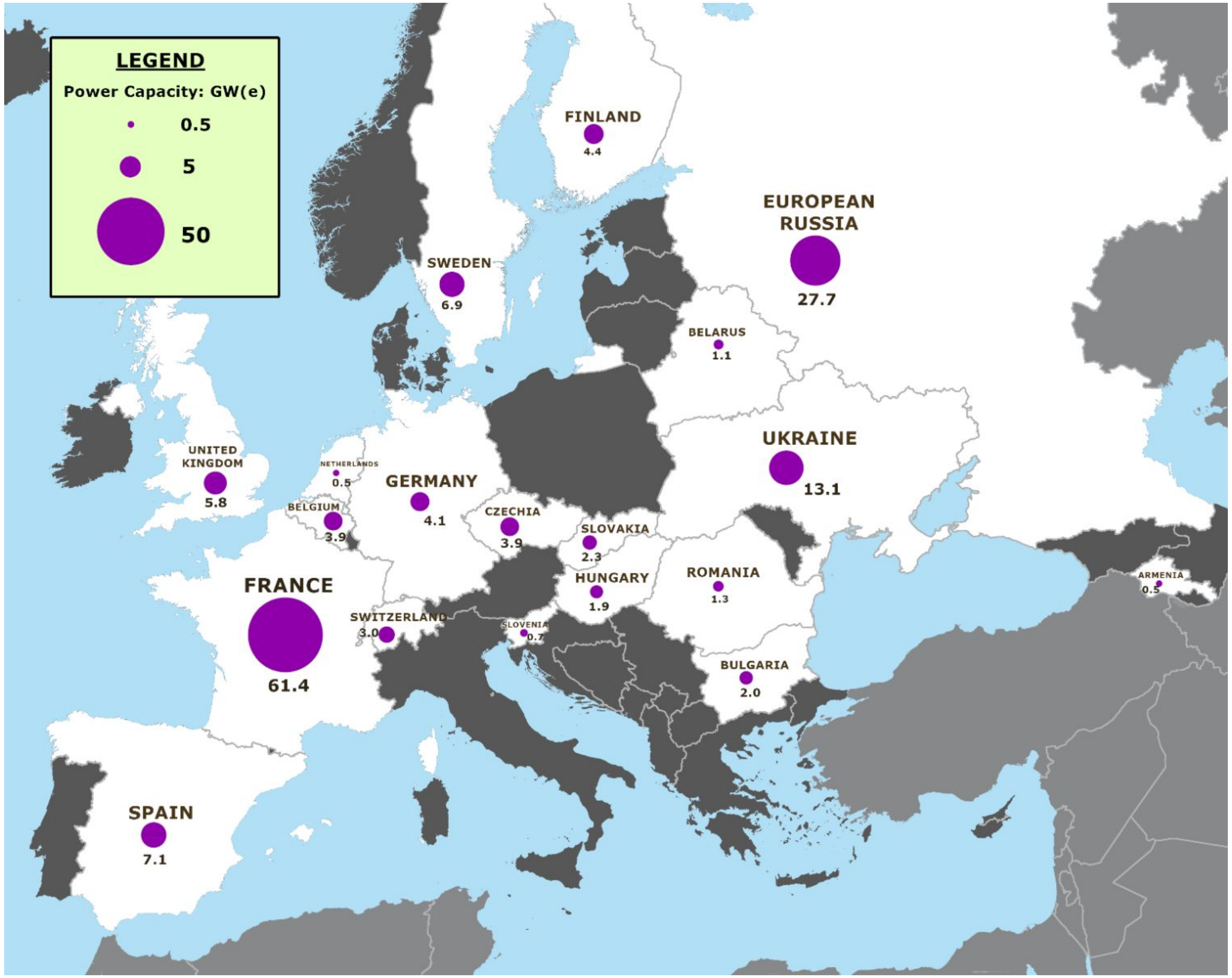


**Figure S7.** A map of continental Europe showing the total nuclear electrical power capacity output per country with an operational commercial nuclear power plant in GW(e). Totalling 170 reactors producing a total of 152 GW(e) of power as of 19/04/2023. Black-shaded countries indicate no current commercial nuclear capacity.

**Table S6.** Induced radioactivity of detectable radioisotopes and their decay. Neat ethylene glycol samples were irradiated with a mixed-field total absorbed dose of 520 kGy (270 kGy from thermal neutrons and 250 kGy from γ rays), producing the following activation products.

| Sample | Radionuclide | Half-life, $T^{1/2}$ / hrs | Concentration / mg kg$^{-1}$ | Sample Activity / Bq | Specific Activity after time, t / Bq g$^{-1}$ | | | | |
|---|---|---|---|---|---|---|---|---|---|
| | | | | at t=0 (After irradiation) | t=0 days | t= 1 day | t= 3days | t=7 days | t=10 days |
| Neat Ethylene Glycol | Br-82 | 35.3 | 0.027 | 248.7 | 104.4 | 65.2 | 25.4 | 9.9 | 4.9 |
| | Na-24 | 14.96 | 1.01 | 16136.1 | 6776.4 | 2228.8 | 241.1 | 26.1 | 4.9 |
| | Sb-124 | 1444.8 | 0.001 | 0.1 | 0.1 | 0.1 | 0.1 | 0.1 | 0.1 |
| | Zn-65 | 5863.2 | 0.191 | 0.3 | 0.1 | 0.1 | 0.1 | 0.1 | 0.1 |
